# P97 Bacterial and fungal secondary infections in COVID-19 patients following immunomodulatory therapy: England, January 2020 to August 2023

**DOI:** 10.1093/jacamr/dlaf230.104

**Published:** 2025-12-04

**Authors:** Chloe Davison, Russell Hope, Colin S Brown, Lance Turtle, Antonia Ho, Sarah M Gerver

**Affiliations:** UK Health Security Agency, London, UK; UK Health Security Agency, London, UK; UK Health Security Agency, London, UK; University of Liverpool, Liverpool, UK; Medical Research Council-University of Glasgow Centre for Virus Research, Glasgow, UK; UK Health Security Agency, London, UK

## Abstract

**Background:**

Immunomodulatory therapy is a key tool to prevent aggressive host-pathogen reactions and associated mortality in severe COVID-19. Secondary infection was detected in 0.6% of COVID-19 patients with immunomodulators versus 0.2% of those with no therapy. However, the impact of this therapy on organisms causing secondary infection in COVID-19 patients is unknown.

**Objectives:**

We aimed to describe the distribution of bacteria and fungi causing secondary infections of COVID-19 patients with and without immunomodulatory therapy.

**Methods:**

COVID-19 episodes from UKHSA’s hospitalized COVID-19 patient dataset (SARS-CoV-2 positive specimen date within 14 days of, or during, hospital admission, 1 January 2020–31 August 2023), bacterial and fungal data from UKHSA’s microbiological datasets (5 December 2019–27 September 2023) and prescribing data from 22 trusts in England which contributed to Electronic Prescribing and Medicines Administrations data (1 January 2020–31 August 2023) were extracted. Medications included were immunomodulators used for COVID-19 therapy administered between 7 days before SARS-CoV-2 positivity to 27 days afterwards or censored to one day before a secondary infection positive specimen collection. Secondary infections were defined as hospitalized COVID-19 patients with a bacterial/fungal infection occurring 2–27 days after SARS-CoV-2 positive specimen date. COVID-19 patients with a bacterial/fungal infection occurring 27 days before to 1 day after SARS-CoV-2 positivity were excluded.

**Results:**

The dataset included 482 COVID-19 episodes with secondary infection, 64.3% of which had no immunomodulators (*n*=310) and 35.7% with immunomodulatory therapy (*n*=172). Within these COVID-19 episodes, 800 bacterial/fungal organism-specific infection episodes were identified during 27 day follow up, the maximum present in one COVID-19 episode was four. There were few demographic differences between those receiving therapy and those without; however, those with immunomodulators tended to have a greater Charlson comorbidity index (CCI) than those without (CCI≥3: 25.0% versus 16.5%, *P*=0.023). Of patients with secondary infection and immunomodulatory therapy, 61.1% had multiple organisms detected versus 48.7% in those receiving no therapy (median: 2 versus 1, *P*=0.0192). Pattern of polymicrobial infection differed by immunomodulator status, for example COVID-19 episodes associated with immunomodulatory therapy were more likely to have a Gram-negative and fungal combination than those without therapy (10.5% versus 4.6%, *P*=0.072). *Enterococcus* spp., *Klebsiella* spp. and *Escherichia* spp. were the most common organisms in all secondary infections (23.0%, 20.7% and 19.3%, respectively) and in the no immunomodulatory therapy group (24.5%, 19.7% and 20.6%, respectively). Whereas the top three in the therapy group were *Klebsiella* spp., *Staphylococcus* spp. and *Pseudomonas* spp. (22.7%, 22.1% and 20.9%, respectively).

**Conclusions:**

COVID-19 patients who receive immunomodulatory therapy have a greater percentage of polymicrobial infections and different genus distribution than those without therapy. Therefore, empirical therapy guidance may need to differ based on immunomodulatory therapy usage for COVID-19 treatment. However, given that patients received immunomodulatory therapy for severe COVID-19, the increased comorbidities and incidence of *Pseudomonas* species may be indicative of patient location (ICU versus non-ICU) confounding the relationship between immunomodulatory therapy and species distribution. At present patients’ ward location is unavailable in the datasets utilized so further work is required to investigate this fully.

